# Supplementary material for: Genome-wide association study in a rat model of temperament identifies multiple loci for exploratory locomotion and anxiety-like traits
Source: Front Genet. 2023 Jan 11;13:1003074. doi: 10.3389/fgene.2022.1003074 (PMC9873817; doi:10.3389/fgene.2022.1003074)
Supplement: Supplementary file 1 [file DataSheet1.PDF]

Supplemental Figure 1. Manhattan plots for all traits

EPM boli

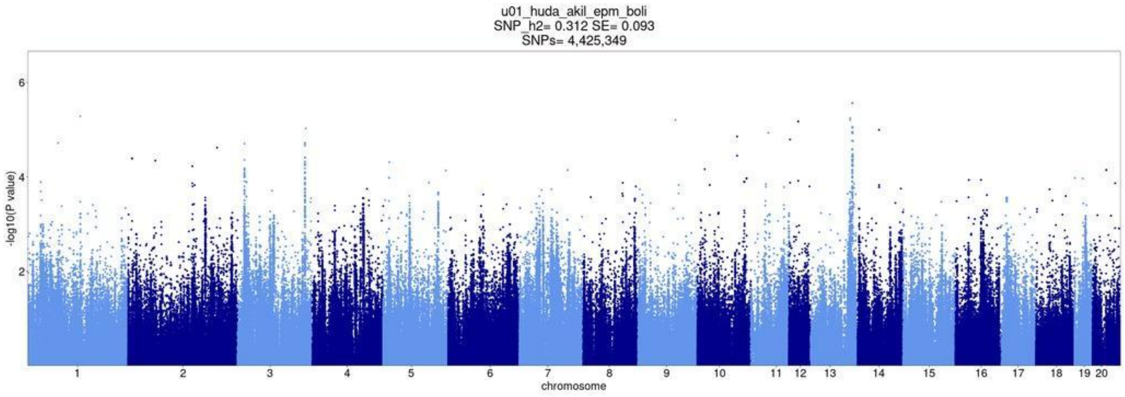

EPM distance traveled

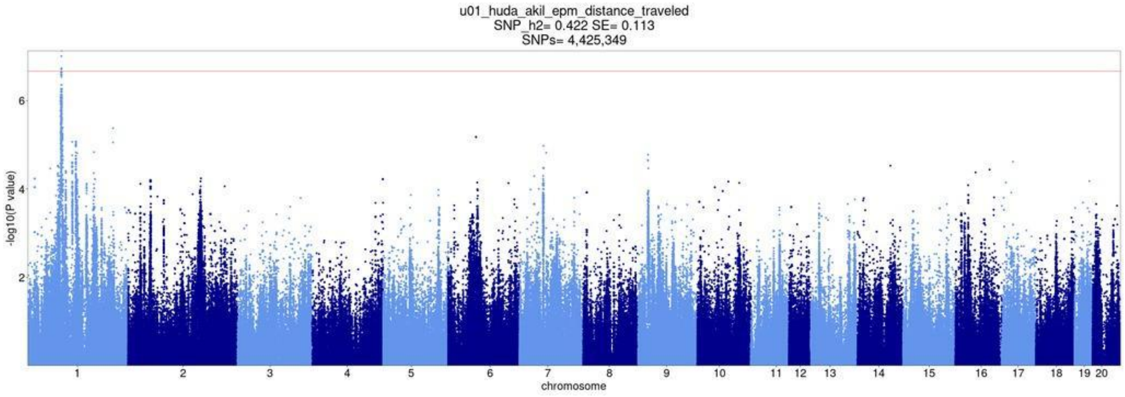

EPM percent time in open arm

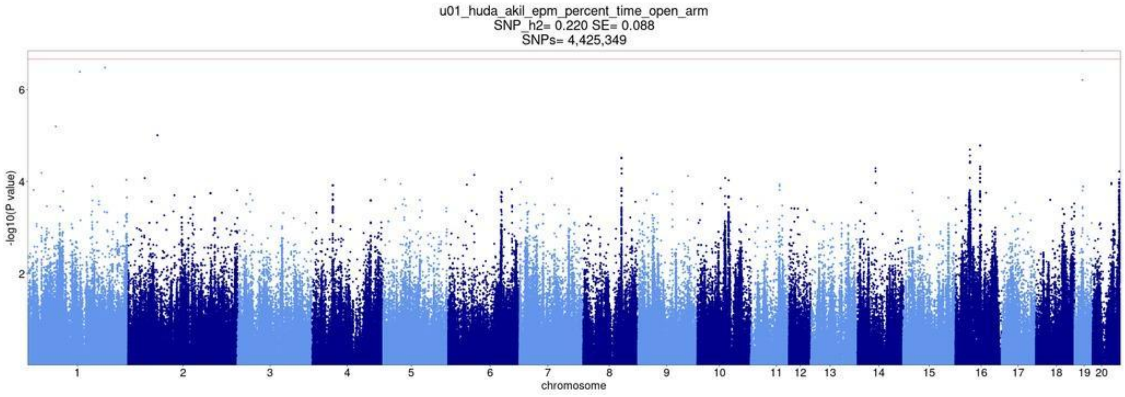

EPM time immobile [s]

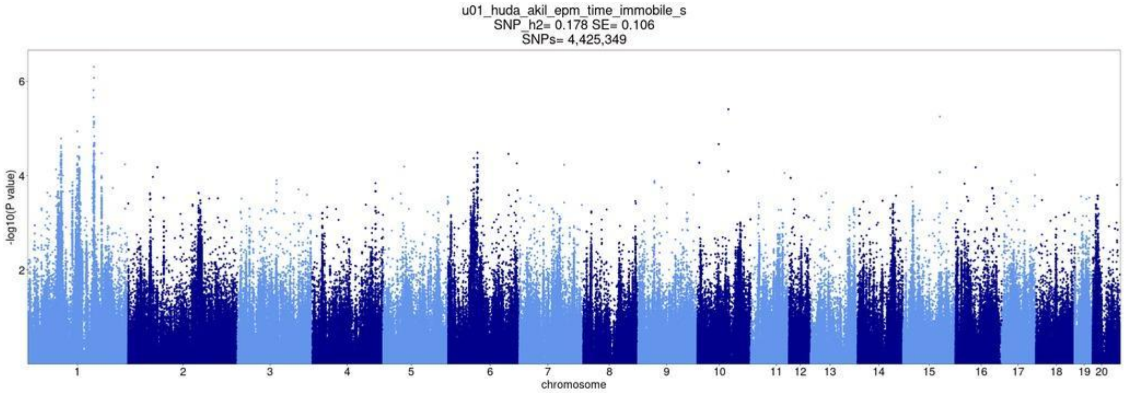

PavCA latency score day6

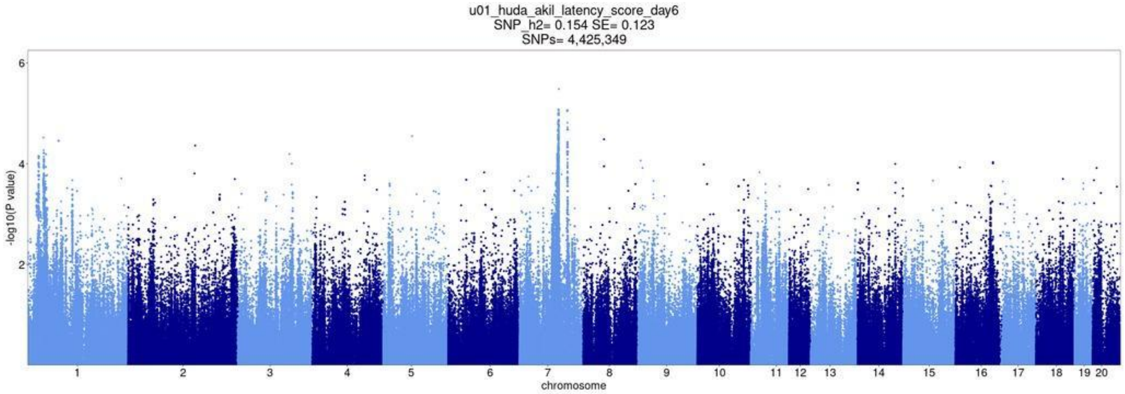

PavCA latency score day7

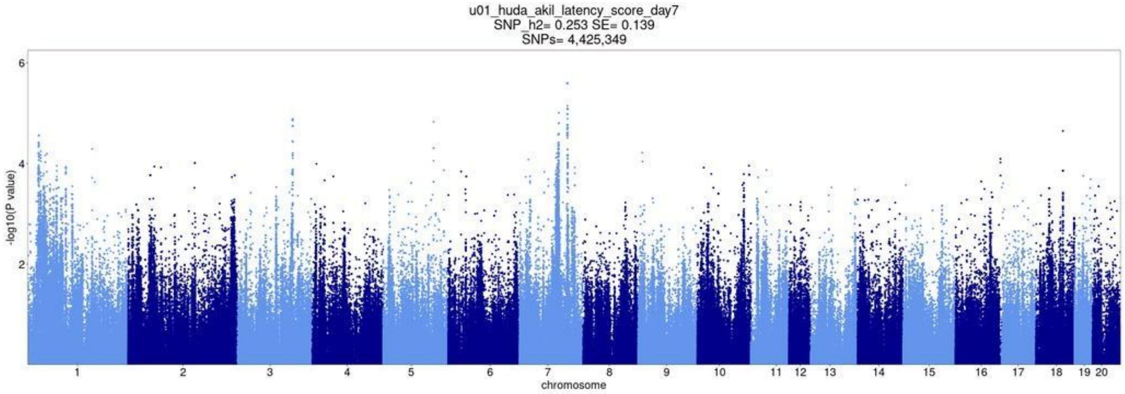

Lateral locomotor score

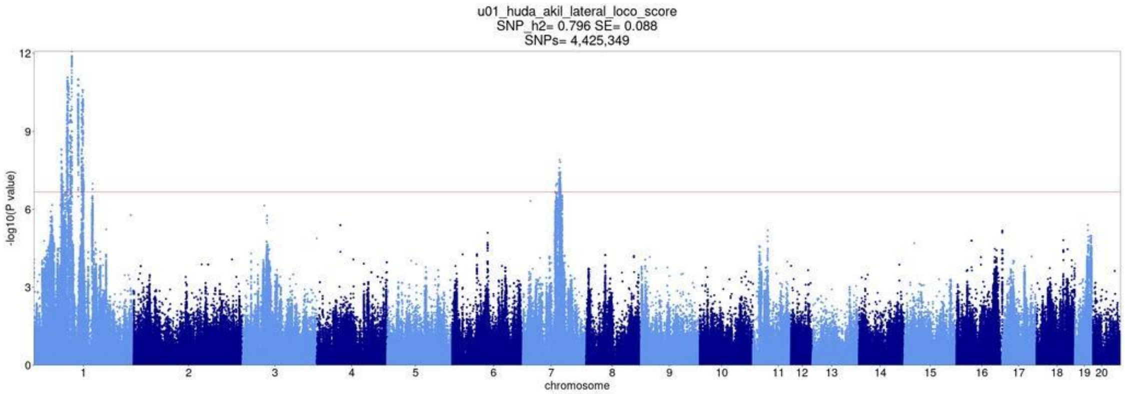

Open field boli

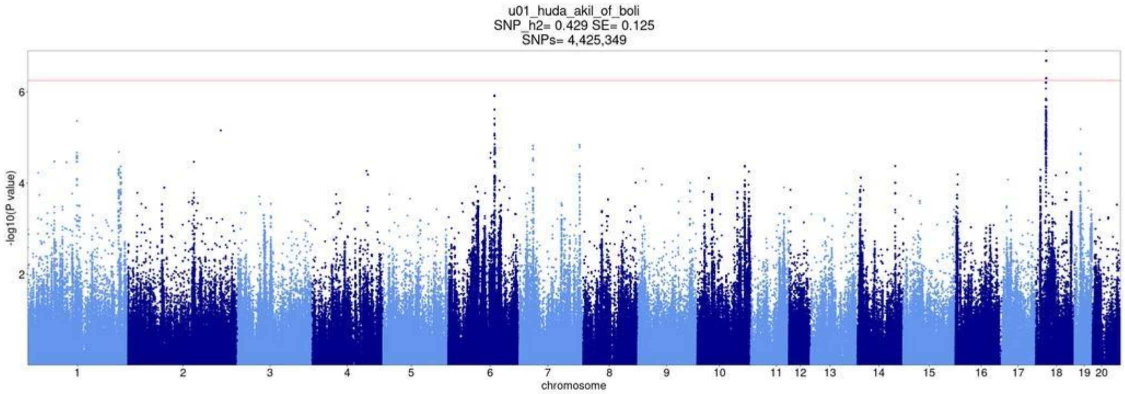

Open field distance traveled

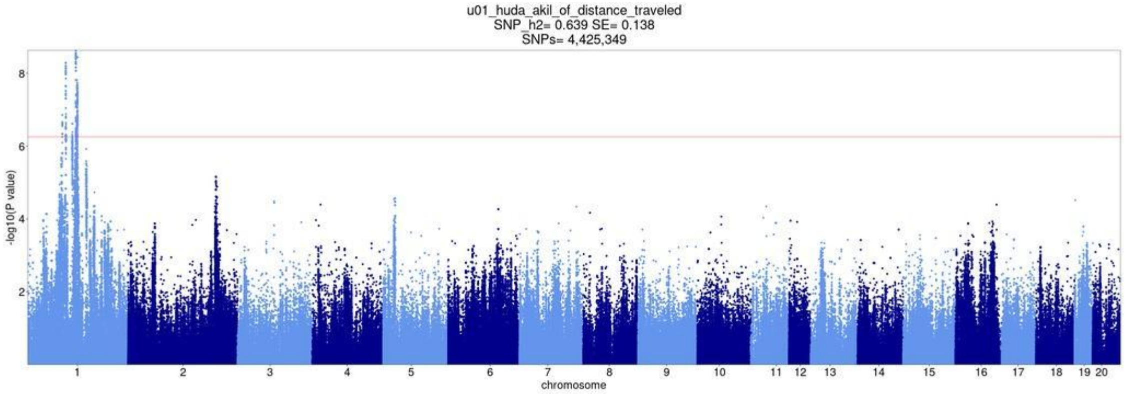

Open field Percent time in center

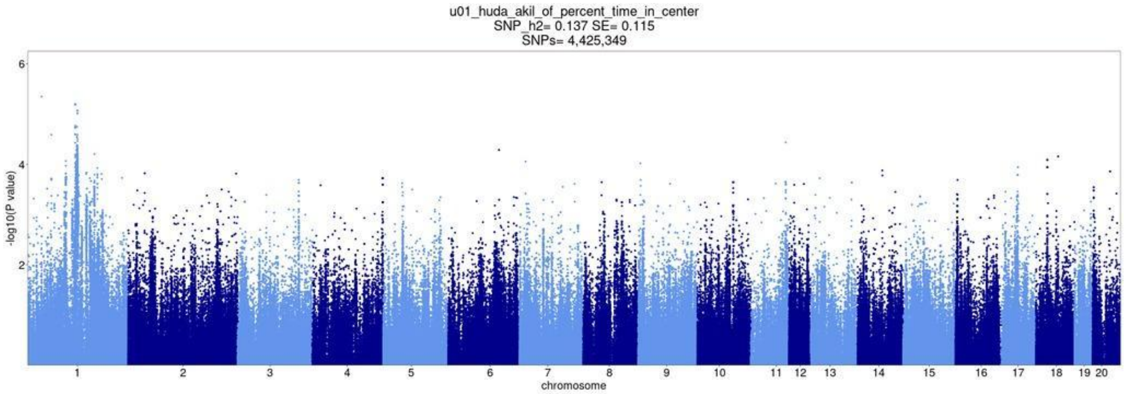

Open field time immobile [s]

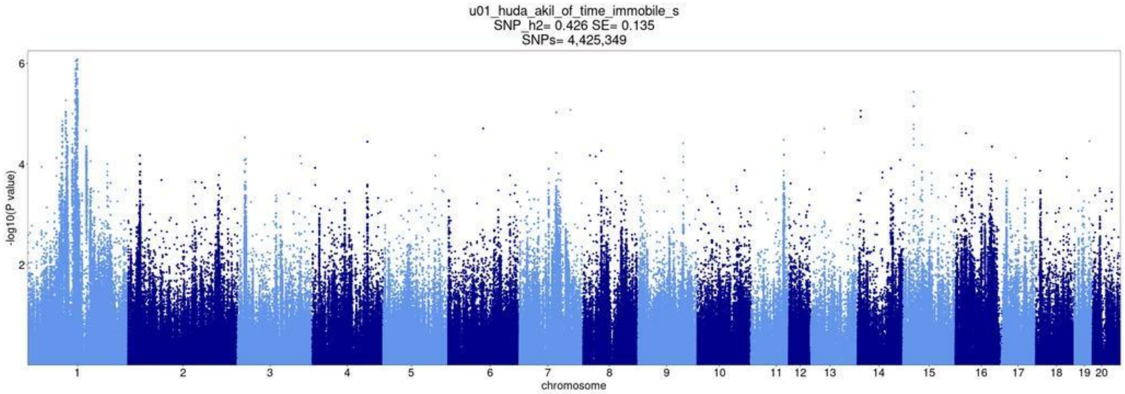

PavCA index day6only

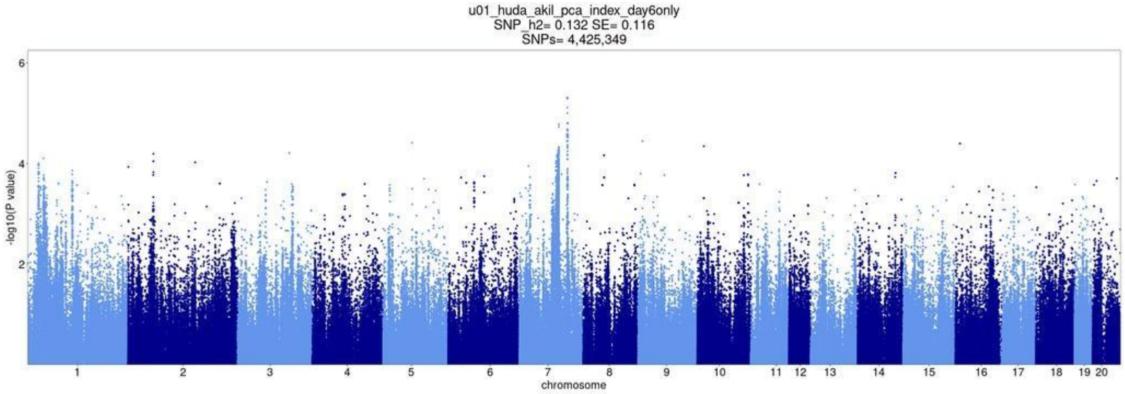

PavCA index day7only

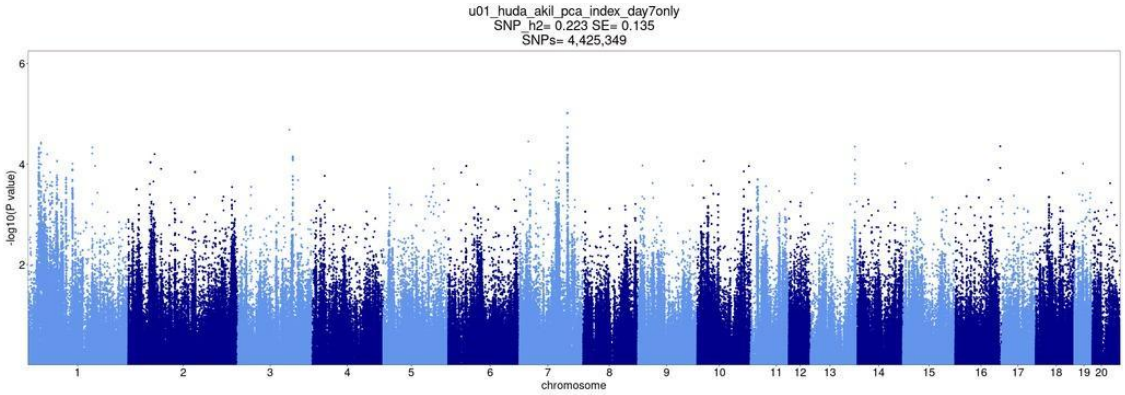

PavCA index days 6and7

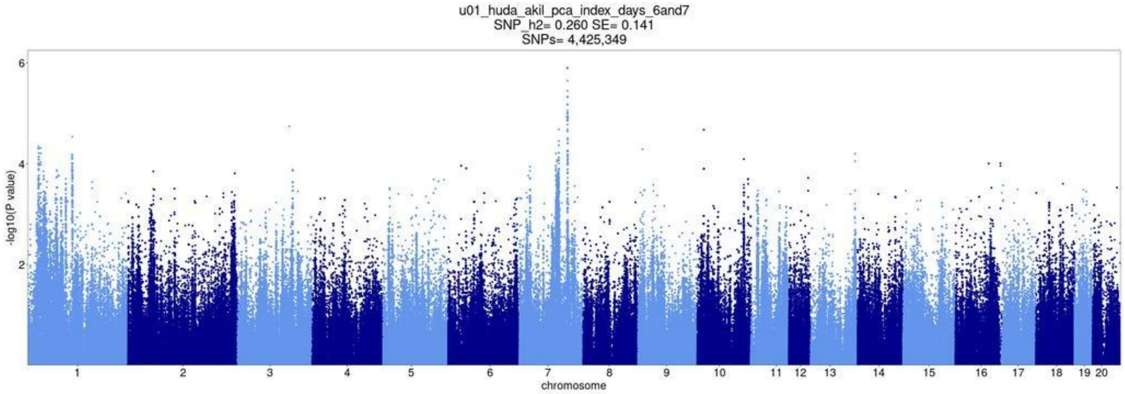

PavCA prob diff day6

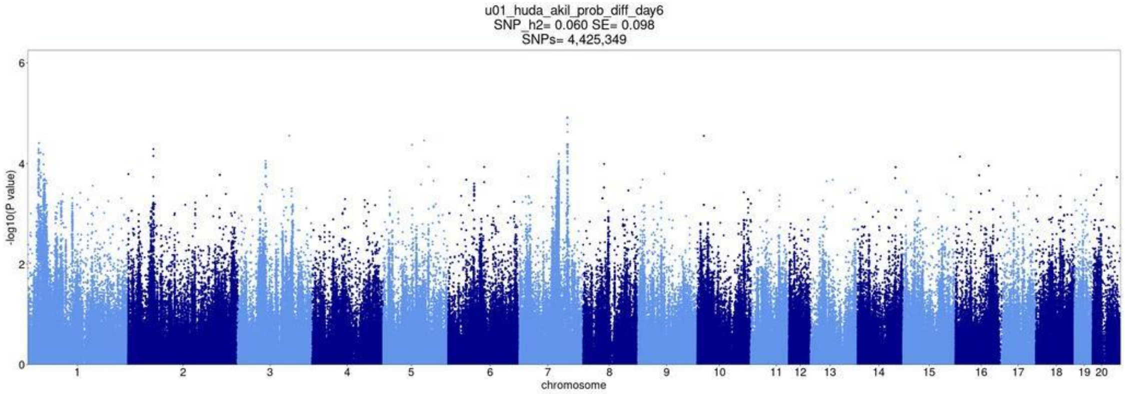

PavCA prob diff day7

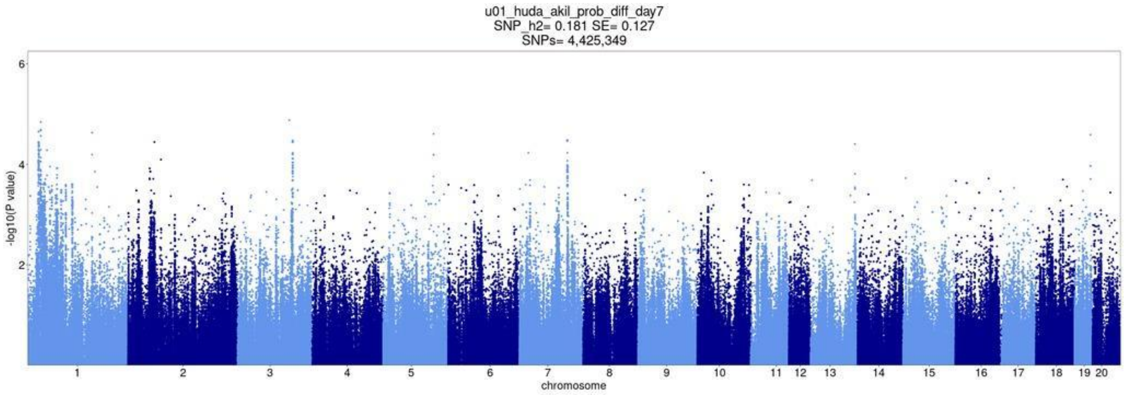

Rearing locomotor score

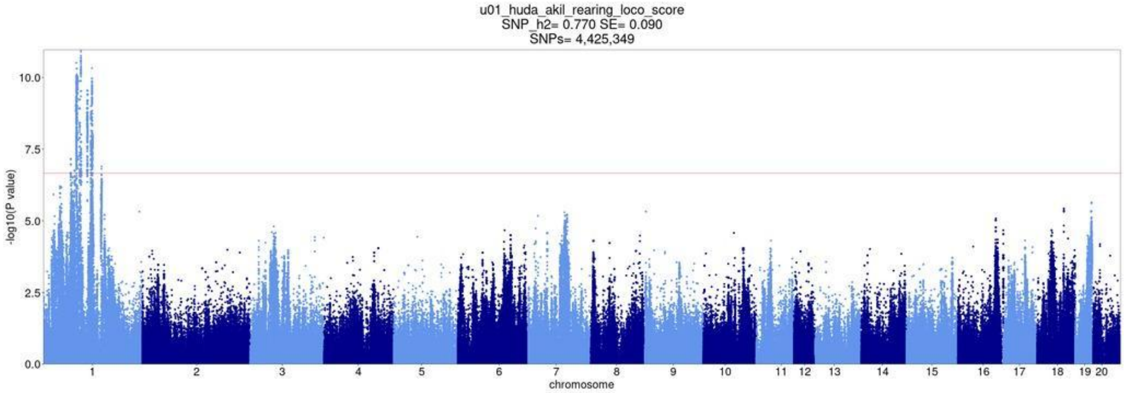

PavCA Response bias day6

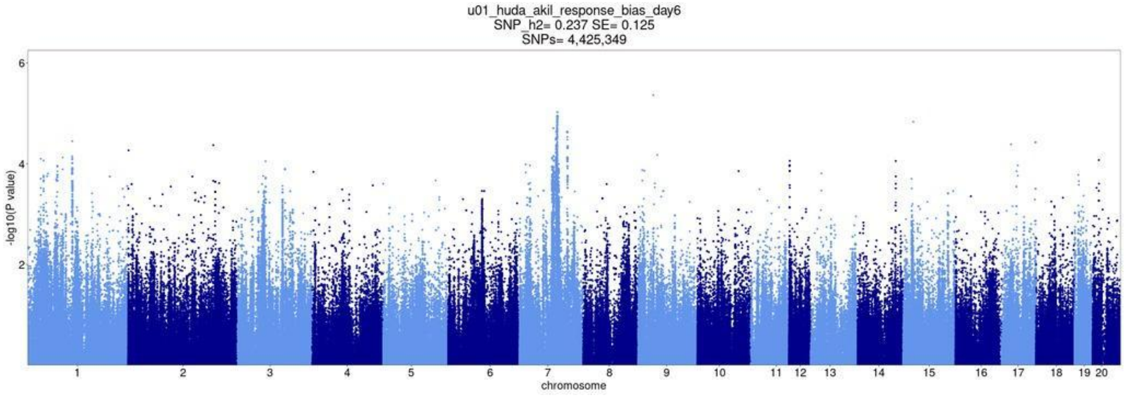

PavCA Response bias day7

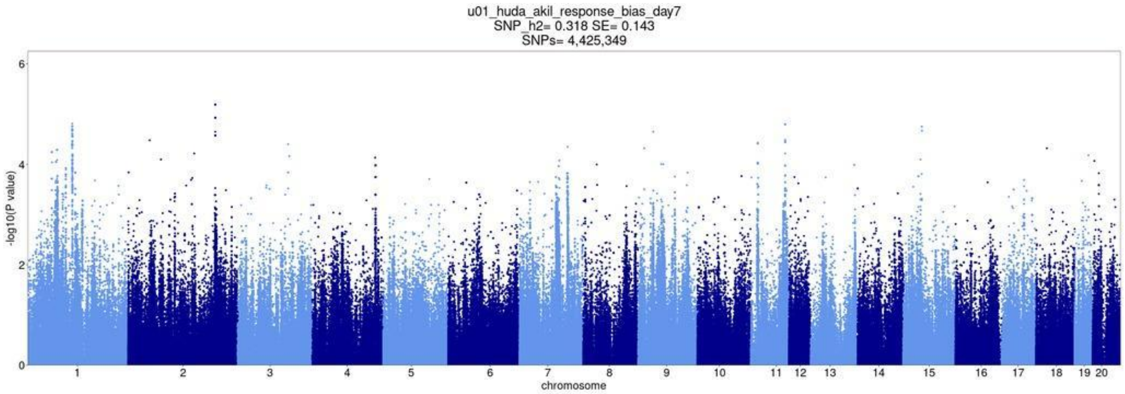

Total locomotor score

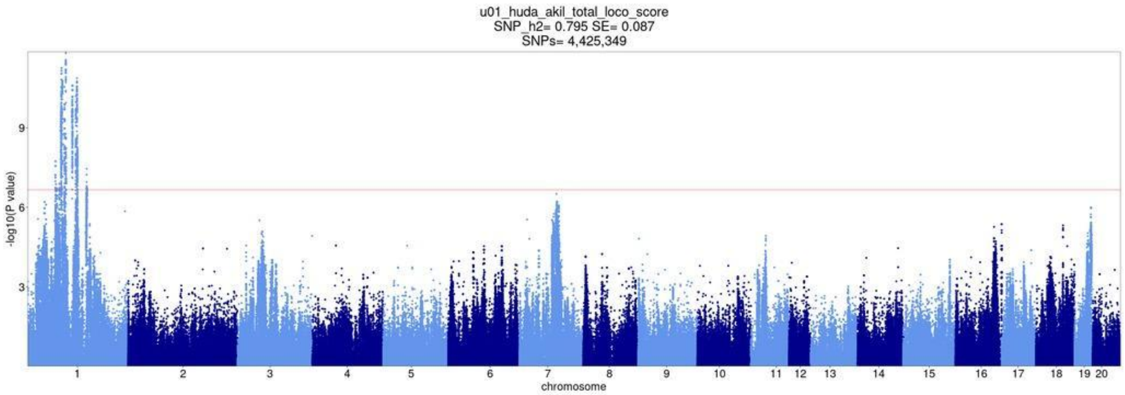

Supplemental Figure 2. Regional association plots for all QTLs.

EPM distance traveled: chr1 94,914,942

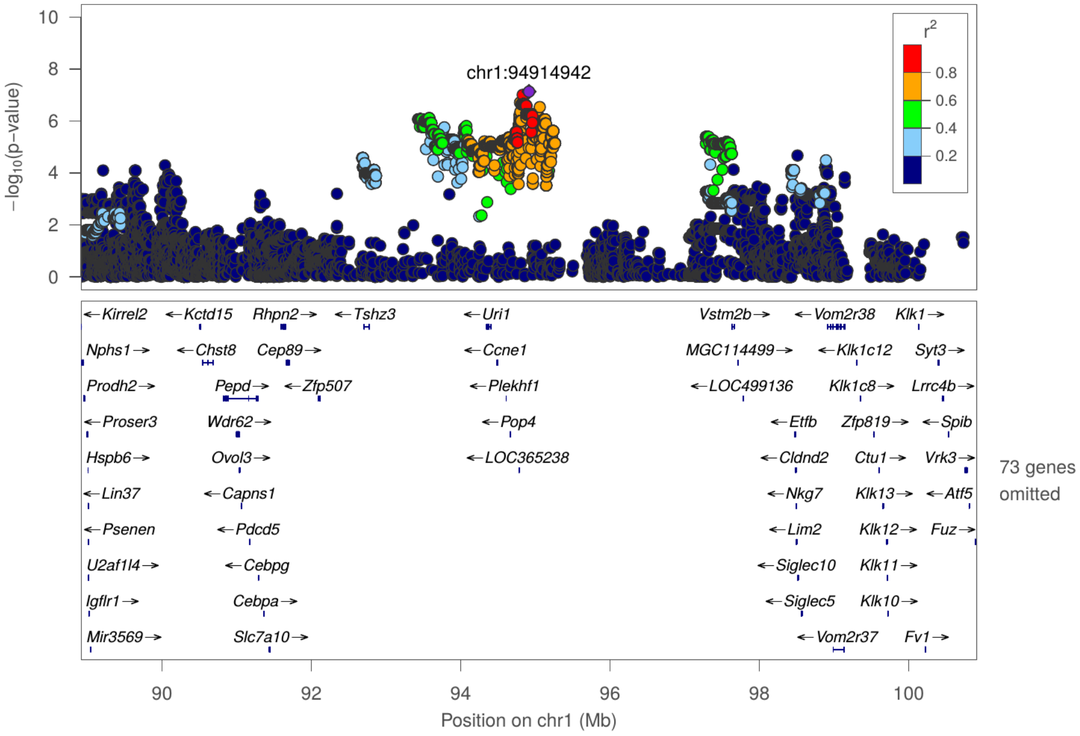

Lateral locomotor score: chr1 107,181,408

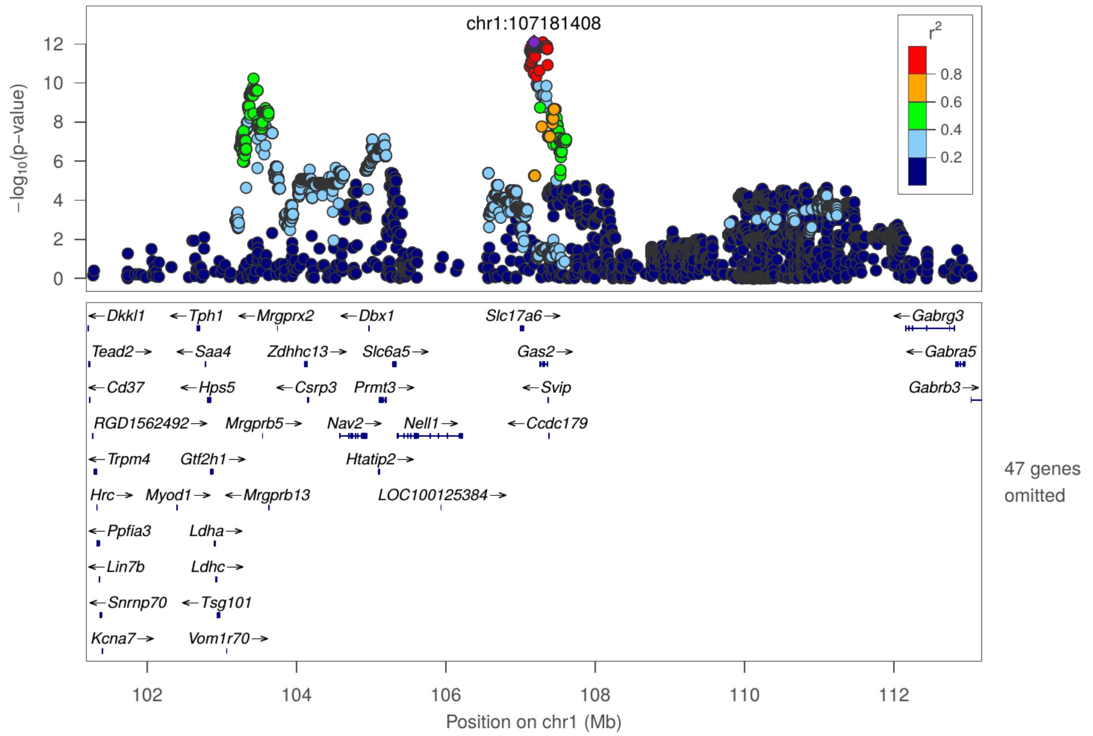

Lateral locomotor score: chr1 95,096,740

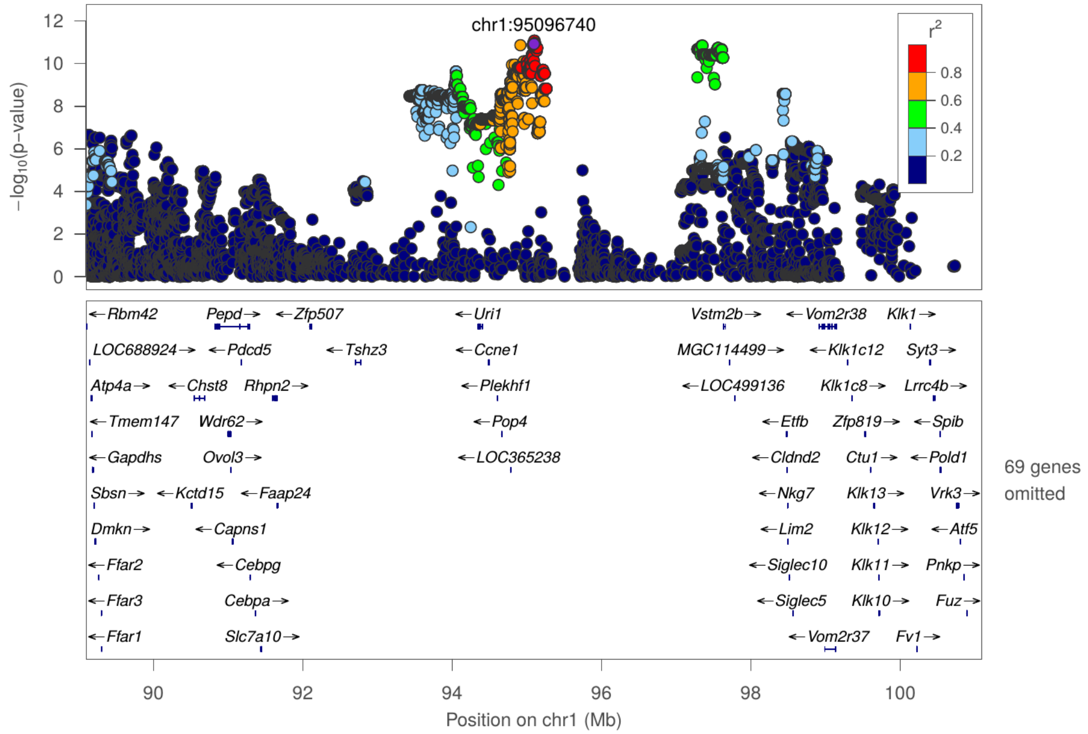

Lateral locomotor score: chr7 84,269,306

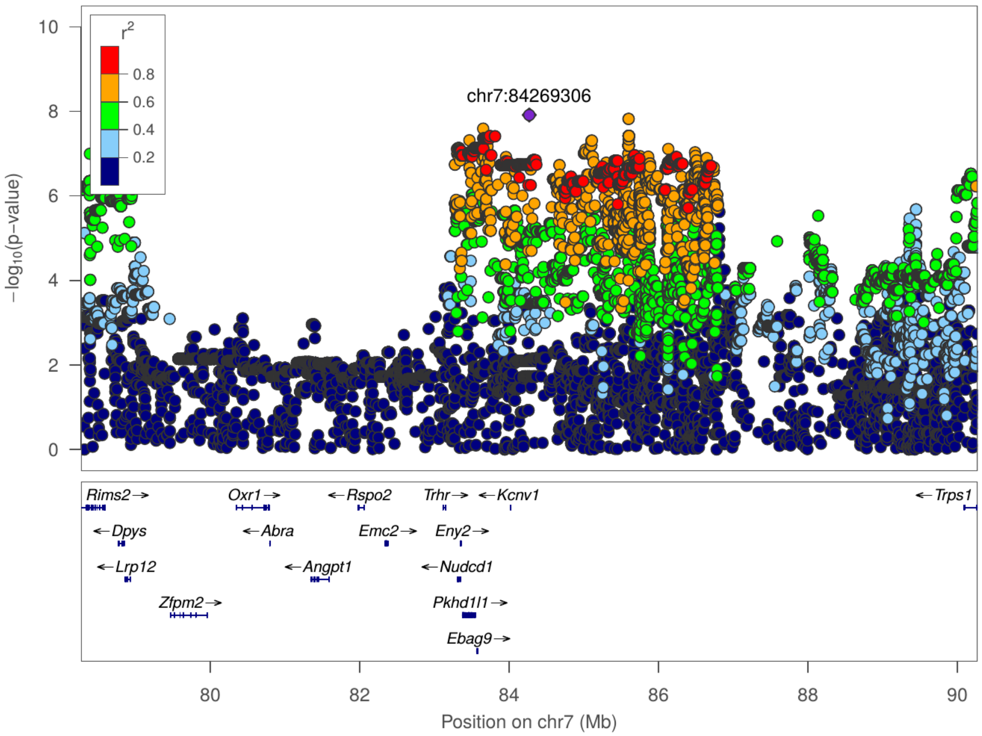

Open field boli: chr18 23,205,496

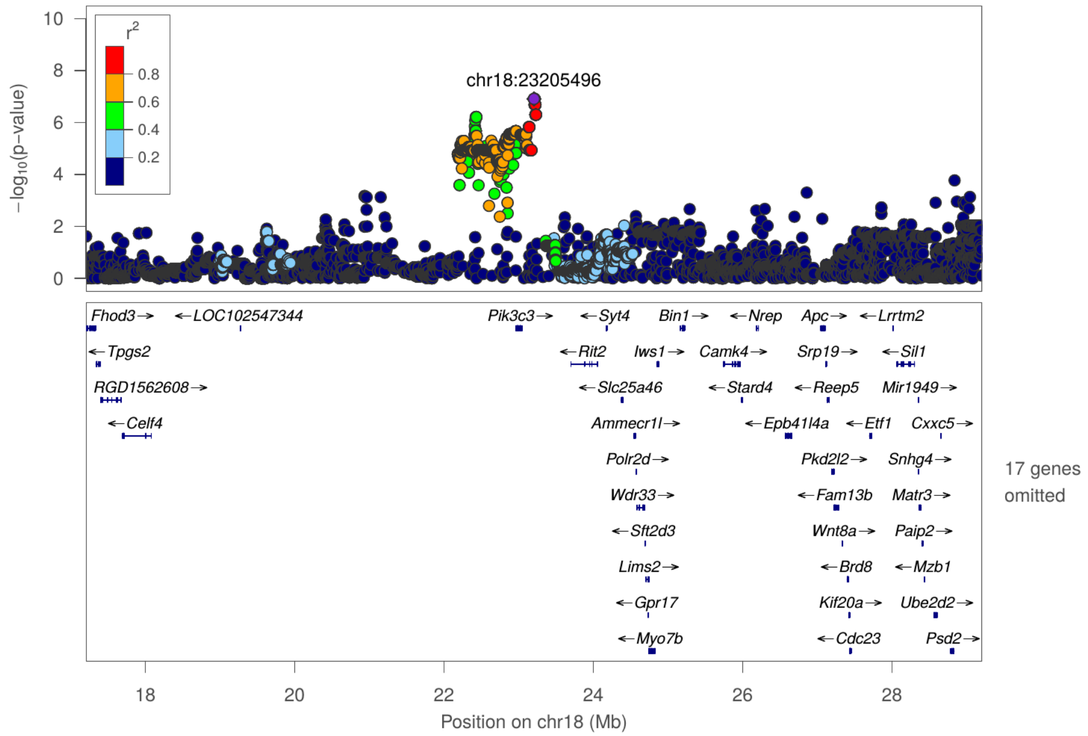

Open field distance traveled : chr1 135,389,579

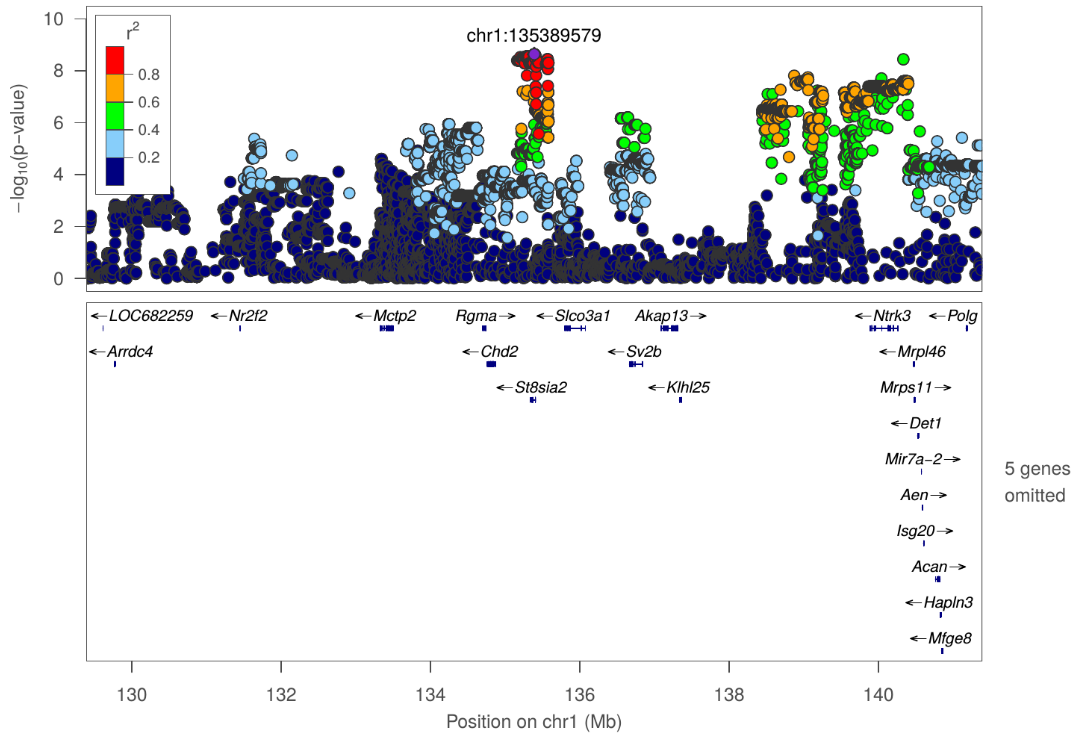

Rearing locomotor score: chr1 107,298,166

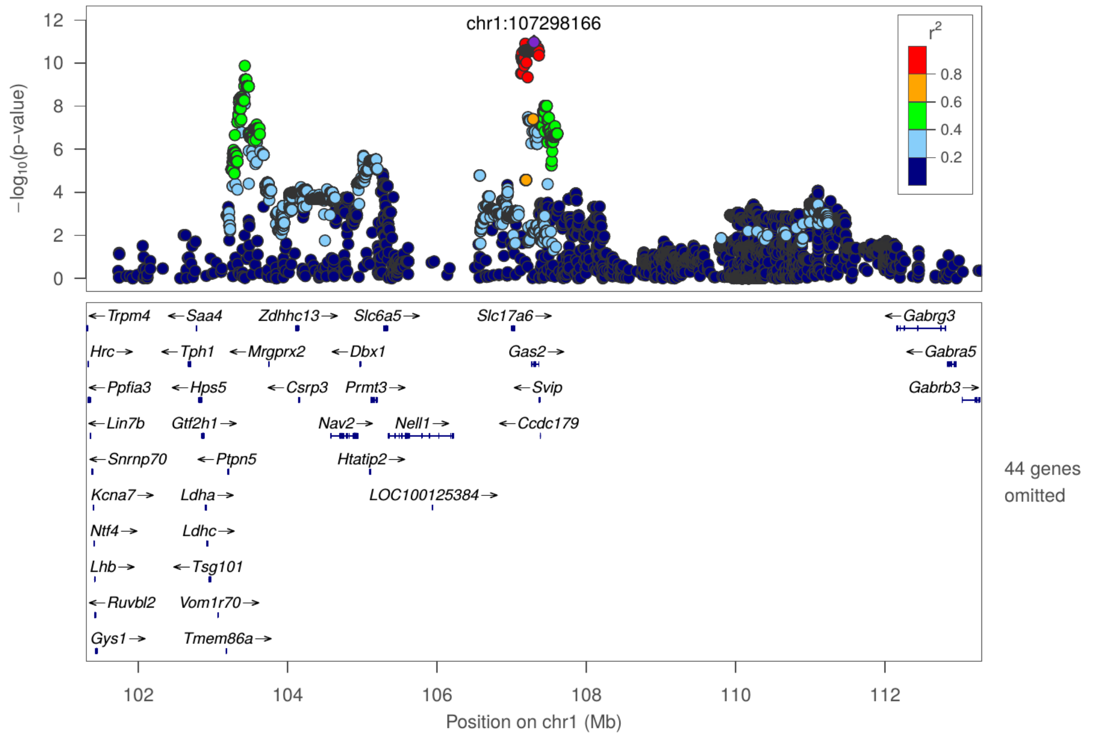

Rearing locomotor score: chr1 95,087,956

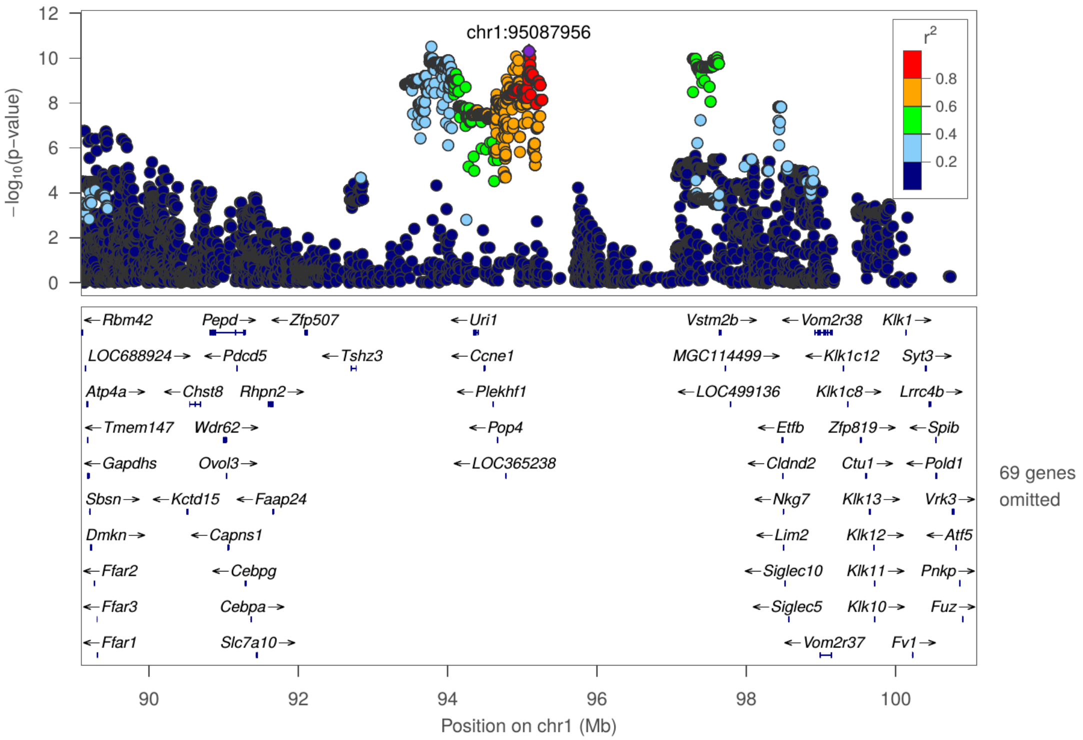

Total locomotor score: chr1 107,298,166

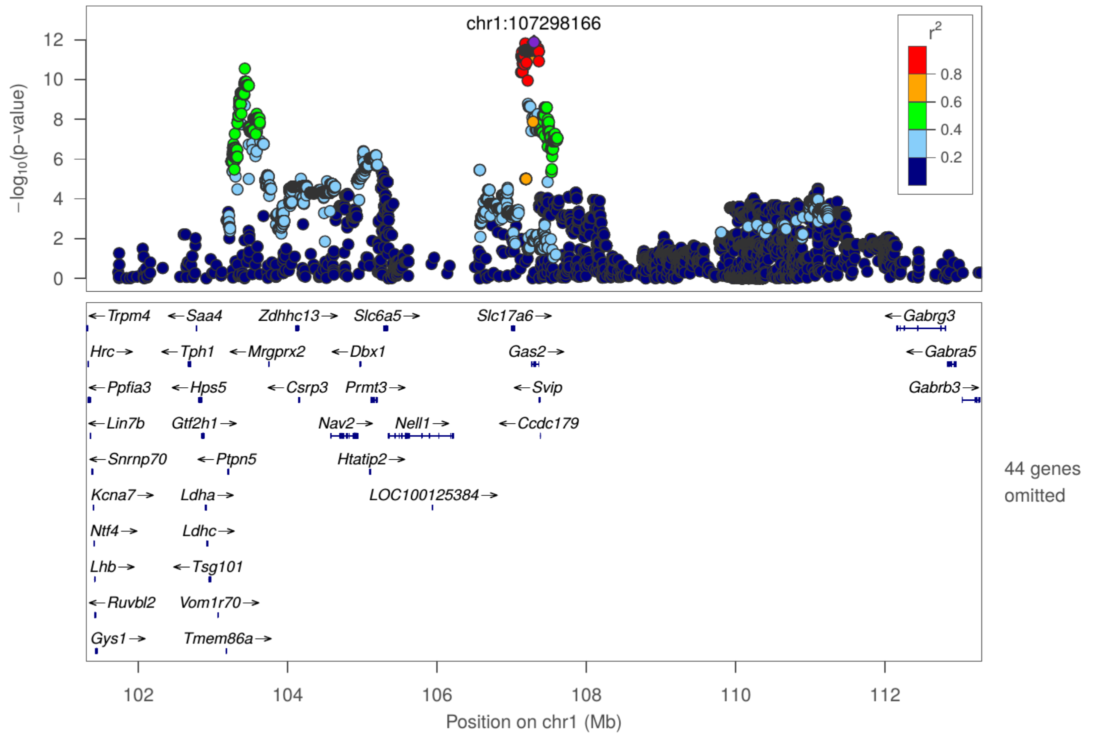

Total locomotor score: chr1 95,096,740

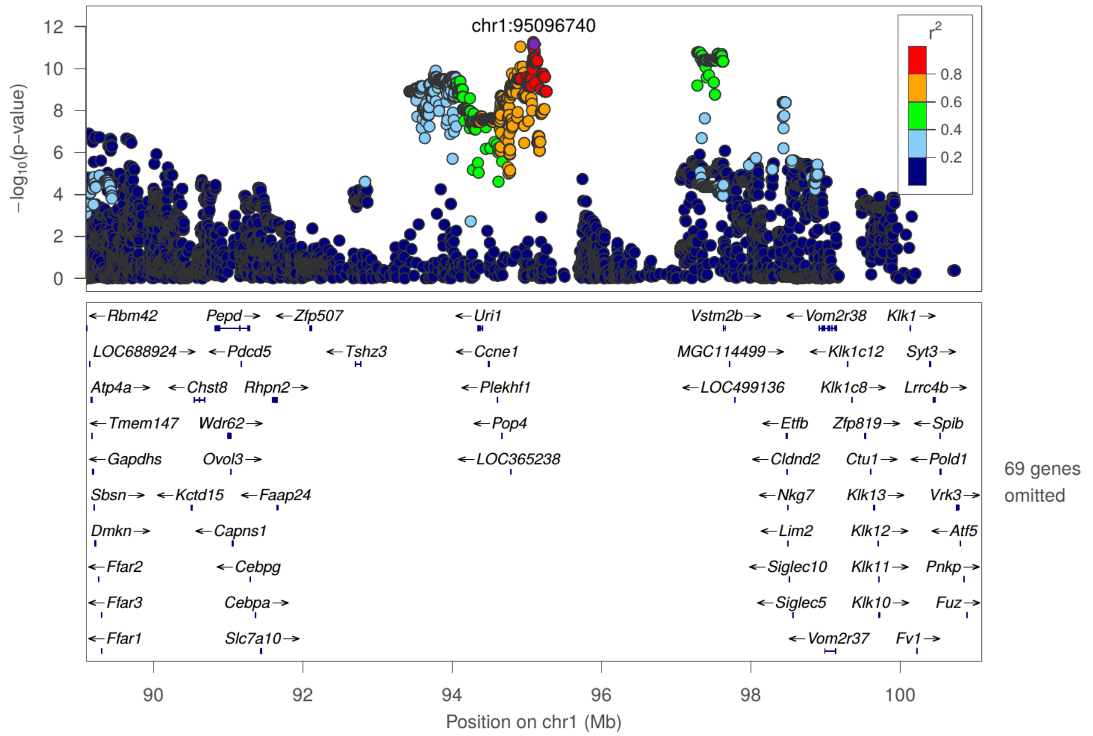

Supplemental Table 1.

Summary statistics for all behavioral traits.

	A. Females						B. Males					
**Traits**	**Stand. dev**	**Mean**	**n**	**Median**	**Minimum**	**Maximum**	**Stand. dev**	**Mean**	**n**	**Median**	**Minimum**	**Maximum**
Lateral locomotor score	114.453	216.682	264	194	34	558	99.874	183.477	275	163.5	19	520
Rearing locomotor score	193.368	351.728	264	324	41	946	168.125	295.657	275	263	21	831
Total locomotor score	300.448	568.411	264	513	75	1461	259.304	479.134	275	432	40	1319
EPM percent time in open arm	10.881	16.759	264	15.67	0	45.03	10.125	11.075	275	8.3	0	46.15
EPM boli	0.648	0.161	264	0	0	5	2.035	1.251	275	0	0	8
EPM distance traveled	430.404	2202.693	264	2168.85	1221.67	3338.77	502.615	1943.112	275	1852.515	1103.76	5208.82
EPM time immobile [s]	18.486	86.597	264	86.29	40.74	147.15	25.146	113.833	275	113.76	63.86	179.08
Open field Percent time in center	2.391	3.145	264	2.467	0	12.467	2.759	2.91	275	2.067	0	14.933
Open field boli	1.433	1.018	264	0	0	6	1.861	1.922	275	2	0	6
Open field distance traveled	580.133	2635.145	264	2634.607	1307.886	4080.152	633.863	2400.69	275	2454.608	293.544	3587.956
Open field time	20.768	61.261	264	58.659	26.827	129.329	30.099	65.846	275	60.661	14.815	230.631

immobile [s]												
PavCA index days 6and7	0.5	0.404	264	0.618	-0.904	0.909	0.518	-0.118	275	-0.221	-0.882	0.848
PavCA Response bias day7	0.684	0.556	264	0.939	-1	1	0.789	-0.182	275	-0.351	-1	1
PavCA prob diff day7	0.534	0.463	264	0.64	-1	1	0.552	-0.047	275	-0.08	-0.92	0.96
PavCA latency score day7	0.341	0.263	264	0.306	-0.715	0.756	0.355	-0.043	275	-0.07	-0.758	0.7
PavCA index day7only	0.506	0.427	264	0.627	-0.905	0.919	0.546	-0.091	275	-0.255	-0.893	0.887
PavCA Response bias day6	0.707	0.479	264	0.895	-1	1	0.757	-0.257	275	-0.52	-1	1
PavCA prob diff day6	0.537	0.402	264	0.56	-0.96	1	0.536	-0.093	275	-0.16	-0.92	0.96
PavCA latency score day6	0.355	0.202	264	0.244	-0.765	0.852	0.337	-0.083	275	-0.116	-0.748	0.602
PavCA index day6only	0.517	0.361	264	0.588	-0.902	0.951	0.529	-0.144	275	-0.366	-0.871	0.817

Supplemental Table 2.

Phenotypic and genetic correlation estimates between all traits.

		Phenotypic correlations		Genetic correlations		
**Trait 1**	**Trait 2**	**rho**	**Pval_pheno**	**rG**	**SE**	**Pval_gen**
**EPM boli**	**EPM dist.**	**-0.17**	**4.23E-04**	**-0.51**	**0.18**	**8.70E-03**
**EPM boli**	**EPM time imm.**	**0.15**	**3.13E-03**	**0.17**	**0.30**	**2.95E-01**
**EPM boli**	**PavCA Lat. D6**	**-0.06**	**3.75E-01**	**0.12**	**0.38**	**3.70E-01**
**EPM boli**	**PavCA Lat. D7**	**0.00**	**9.75E-01**	**0.32**	**0.33**	**1.68E-01**
**EPM boli**	**OF boli**	**NA**	**NA**	**0.45**	**0.29**	**5.77E-02**
**EPM boli**	**OF dist.**	**NA**	**NA**	**-0.36**	**0.26**	**7.95E-02**
**EPM boli**	**OF % time**	**NA**	**NA**	**0.04**	**0.45**	**4.63E-01**
**EPM boli**	**OF time imm.**	**NA**	**NA**	**0.38**	**0.29**	**8.23E-02**
**EPM boli**	**PavCA index D6**	**-0.08**	**2.33E-01**	**0.13**	**0.41**	**3.65E-01**
**EPM boli**	**PavCA index D7**	**-0.01**	**8.89E-01**	**0.24**	**0.34**	**2.35E-01**
**EPM boli**	**PavCA index D6D7**	**-0.04**	**5.97E-01**	**0.17**	**0.32**	**2.99E-01**
**EPM boli**	**PavCA ProbDiff D6**	**-0.06**	**3.83E-01**	**0.39**	**0.64**	**2.37E-01**
**EPM boli**	**PavCA ProbDiff D7**	**0.01**	**8.66E-01**	**0.42**	**0.39**	**1.38E-01**
**EPM boli**	**PavCA Resp. D6**	**-0.06**	**4.18E-01**	**0.37**	**0.34**	**1.34E-01**
**EPM boli**	**PavCA Resp. D7**	**0.00**	**9.83E-01**	**0.24**	**0.32**	**2.24E-01**
**EPM dist.**	**EPM time imm.**	**-0.77**	**0.00E+00**	**NA**	**NA**	**NA**
**EPM dist.**	**PavCA Lat. D6**	**0.26**	**1.51E-04**	**1.00**	**0.29**	**7.12E-05**
**EPM dist.**	**PavCA Lat. D7**	**0.20**	**3.71E-03**	**1.00**	**0.13**	**2.94E-05**
**EPM dist.**	**OF boli**	**NA**	**NA**	**-0.37**	**0.29**	**1.01E-01**
**EPM dist.**	**OF dist.**	**NA**	**NA**	**1.00**	**0.16**	**2.04E-05**
**EPM dist.**	**OF % time**	**NA**	**NA**	**0.64**	**0.45**	**9.34E-02**
**EPM dist.**	**OF time imm.**	**NA**	**NA**	**-0.83**	**0.20**	**4.29E-04**

EPM dist.	PavCA index D6	0.26	1.53E-04	1.00	0.27	6.02E-05
EPM dist.	PavCA index D7	0.22	1.21E-03	1.00	0.14	2.15E-05
EPM dist.	PavCA index D6D7	0.25	2.26E-04	1.00	0.18	1.60E-05
EPM dist.	PavCA ProbDiff D6	0.23	8.14E-04	1.00	0.31	5.41E-04
EPM dist.	PavCA ProbDiff D7	0.21	1.94E-03	0.98	0.15	1.81E-04
EPM dist.	PavCA Resp. D6	0.25	2.09E-04	0.90	0.14	1.39E-04
EPM dist.	PavCA Resp. D7	0.28	4.24E-05	0.91	0.12	3.72E-05
EPM % time	EPM boli	-0.07	1.79E-01	-0.10	0.27	3.50E-01
EPM % time	EPM dist.	0.44	0.00E+00	0.71	0.16	1.37E-03
EPM % time	EPM time imm.	-0.36	5.39E-14	-0.64	0.24	3.34E-02
EPM % time	PavCA Lat. D6	0.11	1.05E-01	0.86	0.37	1.52E-02
EPM % time	PavCA Lat. D7	0.10	1.44E-01	0.78	0.26	7.55E-03
EPM % time	OF boli	NA	NA	0.35	0.38	1.72E-01
EPM % time	OF dist.	NA	NA	0.60	0.26	2.00E-02
EPM % time	OF % time	NA	NA	0.61	0.38	1.08E-01
EPM % time	OF time imm.	NA	NA	-0.69	0.23	7.08E-03
EPM % time	PavCA index D6	0.11	9.74E-02	0.98	0.41	9.15E-03
EPM % time	PavCA index D7	0.10	1.57E-01	0.78	0.29	1.08E-02
EPM % time	PavCA index D6D7	0.12	8.49E-02	0.82	0.27	6.58E-03
EPM % time	PavCA ProbDiff D6	0.10	1.57E-01	1.00	0.59	1.62E-02
EPM % time	PavCA ProbDiff D7	0.11	1.19E-01	0.81	0.29	1.04E-02
EPM % time	PavCA Resp. D6	0.15	3.04E-02	0.55	0.30	4.77E-02
EPM % time	PavCA Resp. D7	0.13	7.04E-02	0.65	0.26	1.85E-02
EPM time imm.	PavCA Lat. D6	-0.25	2.30E-04	-1.00	0.21	7.79E-04

EPM time imm.	PavCA Lat. D7	-0.21	2.86E-03	-1.00	0.19	1.44E-04
EPM time imm.	OF boli	NA	NA	0.12	0.41	3.77E-01
EPM time imm.	OF dist.	NA	NA	-1.00	0.28	6.09E-04
EPM time imm.	OF % time	NA	NA	-1.00	0.62	4.26E-02
EPM time imm.	OF time imm.	NA	NA	1.00	0.25	6.28E-04
EPM time imm.	PavCA index D6	-0.25	2.32E-04	-1.00	0.25	7.30E-04
EPM time imm.	PavCA index D7	-0.22	1.72E-03	-1.00	0.19	2.02E-04
EPM time imm.	PavCA index D6D7	-0.25	3.42E-04	-1.00	0.17	2.38E-04
EPM time imm.	PavCA ProbDiff D6	-0.25	2.83E-04	-1.00	0.70	1.80E-02
EPM time imm.	PavCA ProbDiff D7	-0.23	6.98E-04	-1.00	0.26	4.41E-04
EPM time imm.	PavCA Resp. D6	-0.25	2.64E-04	-1.00	0.29	2.12E-04
EPM time imm.	PavCA Resp. D7	-0.26	1.73E-04	-1.00	0.17	2.43E-05
PavCA Lat. D6	PavCA index D6	0.97	0.00E+00	0.98	0.02	0.00E+00
PavCA Lat. D7	PavCA Lat. D6	0.87	0.00E+00	1.00	0.05	1.15E-02
PavCA Lat. D7	PavCA index D6	0.86	0.00E+00	1.00	0.11	8.51E-02
PavCA Lat. D7	PavCA index D7	0.97	0.00E+00	1.00	0.01	0.00E+00

PavCA Lat. D7	PavCA ProbDiff D6	0.86	0.00E+00	1.00	0.49	1.11E-01
PavCA Lat. D7	PavCA Resp. D6	0.71	0.00E+00	0.93	0.11	8.18E-03
Lat. Loco. Score	EPM boli	-0.10	3.97E-02	-0.15	0.19	2.13E-01
Lat. Loco. Score	EPM dist.	0.40	0.00E+00	0.76	0.10	8.23E-07
Lat. Loco. Score	EPM % time	0.24	5.39E-07	0.70	0.16	4.04E-04
Lat. Loco. Score	EPM time imm.	-0.36	7.80E-14	-1.00	0.13	9.67E-07
Lat. Loco. Score	PavCA Lat. D6	0.41	1.19E-09	0.90	0.27	2.18E-03
Lat. Loco. Score	PavCA Lat. D7	0.38	1.46E-08	0.95	0.18	9.04E-05
Lat. Loco. Score	OF boli	NA	NA	-0.12	0.23	2.85E-01
Lat. Loco. Score	OF dist.	NA	NA	0.88	0.13	2.12E-07
Lat. Loco. Score	OF % time	NA	NA	0.41	0.36	1.19E-01
Lat. Loco. Score	OF time imm.	NA	NA	-0.62	0.19	9.17E-04
Lat. Loco. Score	PavCA index D6	0.43	1.23E-10	1.00	0.31	4.73E-04
Lat. Loco. Score	PavCA index D7	0.39	8.68E-09	0.95	0.20	1.79E-04
Lat. Loco. Score	PavCA index D6D7	0.43	2.01E-10	0.88	0.18	3.01E-04
Lat. Loco. Score	PavCA ProbDiff D6	0.42	4.15E-10	1.00	0.54	1.15E-03

Lat. Loco. Score	PavCA ProbDiff D7	0.40	5.20E-09	1.00	0.28	6.82E-05
Lat. Loco. Score	Rear. Loco. Score	0.88	0.00E+00	0.97	0.01	0.00E+00
Lat. Loco. Score	PavCA Resp. D6	0.48	0.00E+00	1.00	0.13	7.22E-07
Lat. Loco. Score	PavCA Resp. D7	0.43	1.35E-10	1.00	0.16	3.04E-05
Lat. Loco. Score	Tot. Loco. Score	0.94	0.00E+00	0.99	0.01	0.00E+00
OF boli	PavCA Lat. D6	NA	NA	-0.29	0.46	2.64E-01
OF boli	PavCA Lat. D7	NA	NA	0.09	0.42	4.13E-01
OF boli	OF dist.	-0.20	3.68E-03	-0.35	0.22	6.17E-02
OF boli	OF time imm.	0.03	7.08E-01	0.03	0.26	4.51E-01
OF boli	PavCA index D6	NA	NA	-0.06	0.51	4.51E-01
OF boli	PavCA index D7	NA	NA	0.14	0.44	3.70E-01
OF boli	PavCA index D6D7	NA	NA	0.02	0.41	4.84E-01
OF boli	PavCA ProbDiff D6	NA	NA	-0.10	0.67	4.42E-01
OF boli	PavCA ProbDiff D7	NA	NA	0.30	0.49	2.67E-01
OF boli	PavCA Resp. D6	NA	NA	-0.11	0.40	3.82E-01
OF boli	PavCA Resp. D7	NA	NA	0.27	0.38	2.31E-01
OF dist.	PavCA Lat. D6	NA	NA	0.99	0.33	5.99E-03
OF dist.	PavCA Lat. D7	NA	NA	0.91	0.25	2.91E-03
OF dist.	OF time imm.	-0.61	1.72E-23	-0.86	0.08	3.06E-06
OF dist.	PavCA index D6	NA	NA	0.96	0.34	8.57E-03
OF dist.	PavCA index D7	NA	NA	0.93	0.26	3.52E-03
OF dist.	PavCA index D6D7	NA	NA	0.89	0.26	3.54E-03

OF dist.	PavCA ProbDiff D6	NA	NA	1.00	0.47	1.63E-02
OF dist.	PavCA ProbDiff D7	NA	NA	0.96	0.28	4.78E-03
OF dist.	PavCA Resp. D6	NA	NA	0.90	0.30	4.82E-03
OF dist.	PavCA Resp. D7	NA	NA	0.79	0.27	6.28E-03
OF % time	PavCA Lat. D6	NA	NA	0.19	0.80	4.29E-01
OF % time	PavCA Lat. D7	NA	NA	-0.37	0.76	3.52E-01
OF % time	OF boli	-0.09	2.01E-01	-0.37	0.43	1.82E-01
OF % time	OF dist.	0.41	2.56E-10	0.74	0.31	3.49E-02
OF % time	OF time imm.	-0.30	5.05E-06	-0.44	0.33	1.23E-01
OF % time	PavCA index D6	NA	NA	0.12	0.86	4.60E-01
OF % time	PavCA index D7	NA	NA	-0.26	0.79	3.98E-01
OF % time	PavCA index D6D7	NA	NA	0.17	0.67	4.20E-01
OF % time	PavCA ProbDiff D6	NA	NA	-0.01	1.21	4.97E-01
OF % time	PavCA ProbDiff D7	NA	NA	-0.74	0.86	2.59E-01
OF % time	PavCA Resp. D6	NA	NA	0.77	0.62	1.41E-01
OF % time	PavCA Resp. D7	NA	NA	0.53	0.50	1.94E-01
OF time imm.	PavCA Lat. D6	NA	NA	-0.49	0.43	1.35E-01
OF time imm.	PavCA Lat. D7	NA	NA	-0.40	0.36	1.51E-01
OF time imm.	PavCA index D6	NA	NA	-0.40	0.45	2.00E-01
OF time imm.	PavCA index D7	NA	NA	-0.41	0.37	1.53E-01
OF time imm.	PavCA index D6D7	NA	NA	-0.43	0.36	1.26E-01

OF time imm.	PavCA ProbDiff D6	NA	NA	-0.38	0.60	2.73E-01
OF time imm.	PavCA ProbDiff D7	NA	NA	-0.40	0.41	1.82E-01
OF time imm.	PavCA Resp. D6	NA	NA	-0.40	0.37	1.35E-01
OF time imm.	PavCA Resp. D7	NA	NA	-0.44	0.32	9.64E-02
PavCA index D7	PavCA Lat. D6	0.85	0.00E+00	1.00	0.07	2.36E-02
PavCA index D7	PavCA index D6	0.87	0.00E+00	NA	NA	NA
PavCA index D7	PavCA ProbDiff D6	0.86	0.00E+00	1.00	0.47	4.07E-01
PavCA index D7	PavCA Resp. D6	0.75	0.00E+00	0.91	0.12	1.55E-02
PavCA index D6D7	PavCA Lat. D6	0.94	0.00E+00	NA	NA	NA
PavCA index D6D7	PavCA Lat. D7	0.94	0.00E+00	NA	NA	NA
PavCA index D6D7	PavCA index D6	0.97	0.00E+00	NA	NA	NA
PavCA index D6D7	PavCA index D7	0.96	0.00E+00	NA	NA	NA
PavCA index D6D7	PavCA ProbDiff D6	0.95	0.00E+00	1.00	0.07	0.00E+00
PavCA index D6D7	PavCA ProbDiff D7	0.94	0.00E+00	NA	NA	NA
PavCA index D6D7	PavCA Resp. D6	0.84	0.00E+00	0.83	0.14	3.25E-02
PavCA index D6D7	PavCA Resp. D7	0.83	0.00E+00	0.92	0.09	9.06E-03

PavCA ProbDiff D6	PavCA Lat. D6	0.97	0.00E+00	0.98	0.05	0.00E+00
PavCA ProbDiff D6	PavCA index D6	0.99	0.00E+00	1.00	0.04	0.00E+00
PavCA ProbDiff D7	PavCA Lat. D6	0.83	0.00E+00	0.98	0.06	4.89E-02
PavCA ProbDiff D7	PavCA Lat. D7	0.96	0.00E+00	NA	NA	NA
PavCA ProbDiff D7	PavCA index D6	0.85	0.00E+00	1.00	0.07	4.07E-02
PavCA ProbDiff D7	PavCA index D7	0.98	0.00E+00	0.98	0.02	0.00E+00
PavCA ProbDiff D7	PavCA ProbDiff D6	0.85	0.00E+00	NA	NA	NA
PavCA ProbDiff D7	PavCA Resp. D6	0.71	0.00E+00	1.00	0.13	6.24E-03
Rear. Loco. Score	EPM boli	-0.14	4.06E-03	-0.16	0.19	2.02E-01
Rear. Loco. Score	EPM dist.	0.41	0.00E+00	0.73	0.10	3.47E-06
Rear. Loco. Score	EPM % time	0.25	1.70E-07	0.64	0.17	9.78E-04
Rear. Loco. Score	EPM time imm.	-0.43	0.00E+00	-1.00	0.14	2.03E-06
Rear. Loco. Score	PavCA Lat. D6	0.41	6.35E-10	0.92	0.23	4.91E-04
Rear. Loco. Score	PavCA Lat. D7	0.39	7.49E-09	0.90	0.17	8.03E-05
Rear. Loco. Score	OF boli	NA	NA	-0.19	0.23	2.00E-01
Rear. Loco. Score	OF dist.	NA	NA	0.83	0.14	1.41E-06

Rear. Loco. Score	OF % time	NA	NA	0.69	0.36	2.32E-02
Rear. Loco. Score	OF time imm.	NA	NA	-0.58	0.20	2.79E-03
Rear. Loco. Score	PavCA index D6	0.43	1.39E-10	1.00	0.28	1.95E-04
Rear. Loco. Score	PavCA index D7	0.39	1.12E-08	0.90	0.19	2.15E-04
Rear. Loco. Score	PavCA index D6D7	0.43	2.43E-10	0.85	0.18	2.39E-04
Rear. Loco. Score	PavCA ProbDiff D6	0.41	6.06E-10	1.00	0.53	4.77E-04
Rear. Loco. Score	PavCA ProbDiff D7	0.38	1.38E-08	1.00	0.22	1.41E-04
Rear. Loco. Score	PavCA Resp. D6	0.47	0.00E+00	1.00	0.12	6.06E-07
Rear. Loco. Score	PavCA Resp. D7	0.41	1.29E-09	0.91	0.17	1.77E-04
Rear. Loco. Score	Tot. Loco. Score	0.98	0.00E+00	1.00	0.00	0.00E+00
PavCA Resp. D6	PavCA Lat. D6	0.79	0.00E+00	0.74	0.21	8.58E-02
PavCA Resp. D6	PavCA index D6	0.86	0.00E+00	0.79	0.19	8.30E-02
PavCA Resp. D6	PavCA ProbDiff D6	0.82	0.00E+00	1.00	0.37	3.79E-02
PavCA Resp. D7	PavCA Lat. D6	0.68	0.00E+00	0.89	0.17	2.91E-02
PavCA Resp. D7	PavCA Lat. D7	0.77	0.00E+00	0.99	0.08	3.55E-03
PavCA Resp. D7	PavCA index D6	0.74	0.00E+00	1.00	0.15	1.29E-02

PavCA Resp. D7	PavCA index D7	0.85	0.00E+00	0.98	0.06	4.97E-03
PavCA Resp. D7	PavCA ProbDiff D6	0.71	0.00E+00	1.00	0.61	3.81E-02
PavCA Resp. D7	PavCA ProbDiff D7	0.81	0.00E+00	1.00	0.19	8.69E-03
PavCA Resp. D7	PavCA Resp. D6	0.80	0.00E+00	1.00	0.07	1.64E-03
Tot. Loco. Score	EPM boli	-0.13	7.56E-03	-0.17	0.19	1.90E-01
Tot. Loco. Score	EPM dist.	0.42	0.00E+00	0.74	0.10	1.30E-06
Tot. Loco. Score	EPM % time	0.26	7.22E-08	0.67	0.16	5.28E-04
Tot. Loco. Score	EPM time imm.	-0.42	0.00E+00	-1.00	0.14	9.84E-07
Tot. Loco. Score	PavCA Lat. D6	0.42	2.03E-10	0.93	0.24	6.55E-04
Tot. Loco. Score	PavCA Lat. D7	0.40	3.25E-09	0.93	0.17	6.60E-05
Tot. Loco. Score	OF boli	NA	NA	-0.17	0.23	2.24E-01
Tot. Loco. Score	OF dist.	NA	NA	0.86	0.13	4.02E-07
Tot. Loco. Score	OF % time	NA	NA	0.59	0.36	4.48E-02
Tot. Loco. Score	OF time imm.	NA	NA	-0.60	0.19	1.57E-03
Tot. Loco. Score	PavCA index D6	0.44	2.55E-11	1.00	0.28	1.85E-04
Tot. Loco. Score	PavCA index D7	0.40	3.42E-09	0.93	0.19	1.58E-04

Tot. Loco. Score	PavCA index D6D7	0.44	5.24E-11	0.87	0.18	1.91E-04
Tot. Loco. Score	PavCA ProbDiff D6	0.43	1.36E-10	1.00	0.53	4.49E-04
Tot. Loco. Score	PavCA ProbDiff D7	0.40	3.35E-09	1.00	0.24	7.97E-05
Tot. Loco. Score	PavCA Resp. D6	0.49	0.00E+00	1.00	0.12	2.45E-07
Tot. Loco. Score	PavCA Resp. D7	0.43	1.10E-10	0.97	0.16	7.24E-05
Lat. Loco. Score	Lat. Loco. Score	1.00	0.00E+00	1.00	NA	0.00E+00
Rear. Loco. Score	Rear. Loco. Score	1.00	0.00E+00	1.00	NA	0.00E+00
Tot. Loco. Score	Tot. Loco. Score	1.00	0.00E+00	1.00	NA	0.00E+00
PavCA Resp. D6	PavCA Resp. D6	1.00	0.00E+00	1.00	NA	0.00E+00
PavCA Resp. D7	PavCA Resp. D7	1.00	0.00E+00	1.00	NA	0.00E+00
PavCA ProbDiff D6	PavCA ProbDiff D6	1.00	0.00E+00	1.00	NA	0.00E+00
PavCA ProbDiff D7	PavCA ProbDiff D7	1.00	0.00E+00	1.00	NA	0.00E+00
PavCA Lat. D6	PavCA Lat. D6	1.00	0.00E+00	1.00	NA	0.00E+00
PavCA Lat. D7	PavCA Lat. D7	1.00	0.00E+00	1.00	NA	0.00E+00
PavCA index D6	PavCA index D6	1.00	0.00E+00	1.00	NA	0.00E+00
PavCA index D7	PavCA index D7	1.00	0.00E+00	1.00	NA	0.00E+00

PavCA index D6D7	PavCA index D6D7	1.00	0.00E+00	1.00	NA	0.00E+00
EPM % time	EPM % time	1.00	0.00E+00	1.00	NA	0.00E+00
EPM boli	EPM boli	1.00	0.00E+00	1.00	NA	0.00E+00
EPM dist.	EPM dist.	1.00	0.00E+00	1.00	NA	0.00E+00
EPM time imm.	EPM time imm.	1.00	0.00E+00	1.00	NA	0.00E+00
OF % time	OF % time	1.00	0.00E+00	1.00	NA	0.00E+00
OF boli	OF boli	1.00	0.00E+00	1.00	NA	0.00E+00
OF dist.	OF dist.	1.00	0.00E+00	1.00	NA	0.00E+00
OF time imm.	OF time imm.	1.00	0.00E+00	1.00	NA	0.00E+00

Supplemental Table 3. Genotypes of the F_0 rats at moderate impact variants

			bLR-F	bHR-M	bHR-F	bLR-M	bLR-F	bLR-M	bHR-M	bHR-F	bHR-M	bHR-F	bLR-F	bHR-M	bHR-F	bLR-M	bHR-M	bHR-F	bLR-F	bLR-M	bLR-F	bLR-M
Trait	QTL	Candidate gene	5739-JL-0021	5739-JL-0022	5739-JL-0023	5739-JL-0024	5739-JL-0025	5739-JL-0026	5739-JL-0027	5739-JL-0028	5739-JL-0029	5739-JL-0030	5739-JL-0031	5739-JL-0032	5739-JL-0033	5739-JL-0034	5739-JL-0035	5739-JL-0036	5739-JL-0037	5739-JL-0038	5739-JL-0039	5739-JL-0040
EPM distance traveled	chr1: 94 Mb	*Plekhf1*	1/1	0/0	0/1	1/1	1/1	1/1	0/0	0/0	0/0	0/1	1/1	0/1	0/0	1/1	0/1	0/1	0/1	0/1	0/1	1/1
Lateral locomotor score	chr1: 95 Mb	*Plekhf1*	1/1	0/0	0/1	1/1	1/1	1/1	0/0	0/0	0/0	0/1	1/1	0/1	0/0	1/1	0/1	0/1	0/1	0/1	0/1	1/1
Lateral locomotor score	chr7: 84 Mb	*Pkhd1l1*	0/0	1/1	1/1	0/0	0/0	0/0	1/1	1/1	1/1	1/1	0/0	1/1	1/1	0/0	1/1	1/1	0/0	0/0	0/0	0/0
Rearing locomoto	chr1: 95 Mb	*Plekhf1*	1/1	0/0	0/1	1/1	1/1	1/1	0/0	0/0	0/0	0/1	1/1	0/1	0/0	1/1	0/1	0/1	0/1	0/1	0/1	1/1

r score																						
Total locomotor score	chr1: 95 Mb	*Plekhf1*	1/1	0/0	0/1	1/1	1/1	1/1	0/0	0/0	0/0	0/1	1/1	0/1	0/0	1/1	0/1	0/1	0/1	0/1	0/1	1/1

Supplemental Table 4. Genotypes of the F_0 rats at QTL peak markers.

				bLR-F	bHR-M	bHR-F	bLR-M	bLR-F	bLR-M	bHR-M	bHR-F	bHR-M	bHR-F	bLR-F	bHR-M	bHR-F	bLR-M	bHR-M	bHR-F	bLR-F	bLR-M	bLR-F	bLR-M
		**REF**	**ALT**	**5739-JL-0021**	**5739-JL-0022**	**5739-JL-0023**	**5739-JL-0024**	**5739-JL-0025**	**5739-JL-0026**	**5739-JL-0027**	**5739-JL-0028**	**5739-JL-0029**	**5739-JL-0030**	**5739-JL-0031**	**5739-JL-0032**	**5739-JL-0033**	**5739-JL-0034**	**5739-JL-0035**	**5739-JL-0036**	**5739-JL-0037**	**5739-JL-0038**	**5739-JL-0039**	**5739-JL-0040**
**Trait**	**topSNP**	T	C	1/1	0/0	0/1	1/1	1/1	1/1	0/0	0/0	0/0	0/1	1/1	0/1	0/1	1/1	0/1	0/1	0/1	0/1	1/1	1/1
EPM distance traveled	chr1:94,914,942	T	C	0/0	0/0	0/0	0/0	0/0	0/0	0/0	0/1	0/0	0/1	0/0	0/0	0/0	0/0	0/0	0/1	0/0	0/0	0/0	0/0
Lateral locomotor score	chr1:107,181,408	T	G	1/1	0/0	0/0	1/1	1/1	1/1	0/0	0/0	0/0	0/0	1/1	0/1	0/1	1/1	0/0	0/0	1/1	0/1	1/1	1/1
Lateral locomotor score	chr1:95,096,740	C	T	1/1	0/0	0/1	1/1	1/1	1/1	0/0	0/0	0/0	0/1	1/1	0/1	0/0	0/1	0/1	0/1	0/1	0/1	1/1	1/1
Lateral locomotor	chr7:84,269,306	C	T	0/0	1/1	1/1	0/0	0/0	0/0	1/1	0/1	1/1	1/1	0/0	1/1	1/1	0/0	1/1	1/1	0/0	0/0	0/0	0/0

score		T	C																				
OF boli	chr18:23,205,496	T	C	1/1	0/0	1/1	1/1	0/1	0/1	0/0	1/1	0/1	0/1	1/1	0/0	0/0	0/1	0/0	0/0	0/1	0/1	0/1	0/0
OF distance traveled	chr1:135,389,579	G	A	0/0	1/1	0/1	0/0	0/0	0/0	0/1	1/1	1/1	1/1	0/0	1/1	0/1	0/0	1/1	1/1	0/0	0/0	0/0	0/0
Rearing locomotor score	chr1:107,298,166	T	C	1/1	0/0	0/0	1/1	1/1	1/1	0/0	0/0	0/0	0/1	1/1	0/1	0/1	1/1	0/0	0/0	1/1	1/1	1/1	1/1
Rearing locomotor score	chr1:95,087,956	G	A	1/1	0/0	0/1	1/1	1/1	1/1	0/0	0/0	0/0	0/1	1/1	0/1	0/0	0/1	0/1	0/1	0/1	0/1	1/1	1/1
Total locomotor score	chr1:107,298,166	T	C	1/1	0/0	0/0	1/1	1/1	1/1	0/0	0/0	0/0	0/1	1/1	0/1	0/1	1/1	0/0	0/0	1/1	1/1	1/1	1/1
Total locomotor score	chr1:95,096,740	C	T	1/1	0/0	0/1	1/1	1/1	1/1	0/0	0/0	0/0	0/1	1/1	0/1	0/0	0/1	0/1	0/1	0/1	0/1	1/1	1/1

Supplemental Table 5. Relationship between Zhou et al. 2019 and results from the current study.

topSNP from Zhou et al., 2019 study	topSNP from nearby QTL from current study	LD interval of QTL in current study
chr1:50,823,354	Absent	NA
chr1:125,692,364	Absent	NA
chr1:165,902,503	Absent	NA
chr2:61,293,430	Absent	NA
chr3:60,419,606	Absent	NA
chr7:79,179,315	chr7:84,269,306	83,274,530-90,251,798
chr18:81,455,034	Absent	NA
